# Sildenafil normalizes bowel transit in preclinical models of constipation

**DOI:** 10.1371/journal.pone.0176673

**Published:** 2017-04-27

**Authors:** Sarah K. Sharman, Bianca N. Islam, Yali Hou, Margaux Usry, Allison Bridges, Nagendra Singh, Subbaramiah Sridhar, Satish Rao, Darren D. Browning

**Affiliations:** 1 Department of Biochemistry and Molecular Biology, Cancer Research Center, Augusta University, Augusta, Georgia, United States of America; 2 Department of Medicine, Division of Gastroenterology and Hepatology, Augusta University, Augusta, Georgia, United States of America; University Hospital Llandough, UNITED KINGDOM

## Abstract

Guanylyl cyclase-C (GC-C) agonists increase cGMP levels in the intestinal epithelium to promote secretion. This process underlies the utility of exogenous GC-C agonists such as linaclotide for the treatment of chronic idiopathic constipation (CIC) and irritable bowel syndrome with constipation (IBS-C). Because GC-C agonists have limited use in pediatric patients, there is a need for alternative cGMP-elevating agents that are effective in the intestine. The present study aimed to determine whether the PDE-5 inhibitor sildenafil has similar effects as linaclotide on preclinical models of constipation. Oral administration of sildenafil caused increased cGMP levels in mouse intestinal epithelium demonstrating that blocking cGMP-breakdown is an alternative approach to increase cGMP in the gut. Both linaclotide and sildenafil reduced proliferation and increased differentiation in colon mucosa, indicating common target pathways. The homeostatic effects of cGMP required gut turnover since maximal effects were observed after 3 days of treatment. Neither linaclotide nor sildenafil treatment affected intestinal transit or water content of fecal pellets in healthy mice. To test the effectiveness of cGMP elevation in a functional motility disorder model, mice were treated with dextran sulfate sodium (DSS) to induce colitis and were allowed to recover for several weeks. The recovered animals exhibited slower transit, but increased fecal water content. An acute dose of sildenafil was able to normalize transit and fecal water content in the DSS-recovery animal model, and also in loperamide-induced constipation. The higher fecal water content in the recovered animals was due to a compromised epithelial barrier, which was normalized by sildenafil treatment. Taken together our results show that sildenafil can have similar effects as linaclotide on the intestine, and may have therapeutic benefit to patients with CIC, IBS-C, and post-infectious IBS.

## Introduction

Irritable bowel syndrome (IBS) is a functional gastrointestinal disorder characterized by altered bowel habits and abdominal pain that adversely affect quality of life. IBS is sub-classified as constipation-predominant (IBS-C), diarrhea-predominant (IBS-D), or mixed symptom (IBS-M). There is no cure for IBS, and current treatment strategies often require patients to take multiple medications to control their symptoms [1]. Bulking agents, laxatives, and anti-diarrheals are prescribed to help normalize alterations in bowel habits, while tricyclic antidepressants and antispasmodics aim to minimize visceral pain associated with the disease [2]. The variable effectiveness of this symptom-targeted approach to IBS treatment underscores the need for alternatives.

The major clinical hallmarks of IBS are alterations in intestinal motility, secretion, and visceral sensation. Although the underlying cause of IBS is unknown, numerous studies indicate an important role for 5-hydroxytryptamine (5-HT, serotonin) [3,4]. Alterations in serotonin levels and enterochromaffin cell densities are common in patients with IBS, and serotonin has an established role in the regulation of intestinal motility and enteric nociception [5,6,7]. In support of this idea, targeting the serotonin system pharmacologically has had some success in the clinic. Selective serotonin reuptake inhibitors (SSRIs) alter motility and decrease visceral pain but are not convincingly beneficial for IBS [8]. 5HT-4 receptor agonists such as prucalopride stimulate peristaltic reflex thereby accelerating gastrointestinal transit and inhibiting visceral hypersensitivity [9,10,11]. In addition, 5HT-3 receptor antagonists delay transit but also alleviate visceral pain in IBS-D patients [12]. While these drugs are successful for some patients, they do not resolve all IBS symptoms.

It is well-established that cyclic guanosine monophosphate (cGMP) activates secretion in the intestine by regulating ion channels such as the cystic fibrosis transmembrane conductance regulator (CFTR) [13,14]. The intestinal hormones guanylin and uroguanylin increase cGMP by binding and activating epithelial guanylyl cyclase-C (GC-C). GC-C agonists are a novel class of drugs that have emerged for the treatment of IBS-C and CIC. Linaclotide is currently the only FDA-approved member of this family and increases cGMP levels in the intestinal epithelium by stimulating GC-C receptors [15,16]. Increased fluid secretion in response to linaclotide is likely to be central to the therapeutic effect of the drug on constipation [17]. However, linaclotide has also been shown to affect neuromuscular function and reduce visceral pain in human patients as well as in rodents, suggesting an additional role for cGMP signaling in the gut [18,19]. The promising effects of linaclotide on constipation underscore the potential importance of cGMP signaling in the treatment of gastrointestinal diseases. Preclinical studies show that mice deficient in cGMP signaling components have intestinal barrier dysfunction, higher levels of proliferation and apoptosis, and reduced differentiation of secretory cells in the intestinal epithelium [20,21,22]. While the mechanism behind the regulation of secretion by cGMP is understood, the mechanistic details of the cGMP effect on motility or homeostasis are far from clear.

It was recently reported that blocking cGMP degradation using the phosphodiesterase 5 (PDE-5) inhibitor vardenafil led to increased cGMP and altered homeostasis in the intestinal epithelium of mice [23]. Since both PDE-5 inhibitors and GC-C agonists such as linaclotide increase cGMP in the intestinal epithelium, these drugs may have similar therapeutic value for IBS-C and CIC. Because PDE-5 inhibitors such as sildenafil are safe for extended use in pediatric patients, this class of drugs might offer an alternative to linaclotide in children under 17 for which linaclotide is contraindicated. The present study tested the ability of sildenafil to regulate bowel transit in preclinical models of constipation. Sildenafil had no effect on intestinal transit or fecal water content in healthy mice. However, in dextran sulfate sodium (DSS)-recovery model of constipation, both sildenafil and linaclotide normalized transit time and fecal water content that are elevated in the model. In addition, sildenafil and linaclotide were equally effective at normalizing transit time in a model of opioid-induced constipation (OIC). Results shown here suggest that PDE-5 inhibitors might be an effective treatment for IBS-C and post-inflammatory IBS.

## Methods

### Animals and drug administration

This study was carried out in compliance with recommendations in the Guide for the Care and Use of Laboratory Animals of the National Institutes of Health, and all animal procedures were carried out under protocols (2010–0201, 2011–0318) approved by the Institutional Animal Care and Use Committee (IACUC) at Augusta University. Euthanasia of mice was by carbon dioxide asphyxiation and thoracotomy, and every effort was made to minimize stress and suffering. Six to eight week old CD1 mice were purchased from Harlan Laboratories (Indianapolis, IN, USA) and C57/BL6 mice were purchased from Jackson Laboratories (Bar Harbor, ME, USA). Mice were housed under standard conditions with unlimited access to food and water. Male mice were used for IHC, cGMP assays, and for the inflammation-recovery model. Both male and female mice were used for intestinal transit assays. Pharmaceutical grade sildenafil citrate was ground with a mortar and pestle and stored as a suspension in di-H_2_O at -20°C. Based upon mean water intake per animal, stock sildenafil was added to the drinking water at concentrations that approximated doses of 0.36–17 mg/kg daily per mouse. Unless otherwise stated, the dose of sildenafil administered in the drinking water was equivalent to 5.7 mg/kg per day. For oral gavage and intraperitoneal injection, mice were administered 100μl of 1.4 mg/ml sildenafil. For intraperitoneal injections of vardenafil, pharmaceutical grade vardenafil was stored frozen in DMSO at 1.5 mg/ml and diluted 1/10 in PBS prior to injection (100μl twice daily). Linaclotide was prepared by grinding the contents of Linzess^®^ capsules in di-H_2_O using a tissue homogenizer and stored in aliquots at -80°C. Mice were gavaged daily with 100μl of the linaclotide suspension at a concentration of 2.07 μg/ml. Loperamide HCl (Sigma-Aldrich) was dissolved in di-H_2_O at a concentration of 2.5 mg/ml. Mice were gavaged with 100μl of loperamide one hour prior to the intestinal transit assay. All other reagents were purchased from Fisher Scientific (Waltham, MA, USA).

### IHC and cGMP measurements

Animal tissues were processed for histological analysis as previously described [20]. Briefly, tissues were fixed with 10% formaldehyde, embedded in paraffin blocks, and sectioned by the Augusta University histology core. The tissues were probed using antibodies to CgA (1:200; Immunostar, Hudson, WI, USA), Ki-67 (1:100; Dako Cytomation, Carpinteria, CA, USA), cleaved caspase-3 (1:500; Cell Signaling, Danvers, MA, USA), and 5-HT (1:5000; Immunostar, Hudson, WI, USA). Visualization of Ki-67 and cleaved caspase antibodies was done using the ImmunoCruz ABC kit (Santa Cruz Biotechnology, Dalls, TX, USA). To ensure the specificity of the 5-HT and CgA antibodies, we used a commercially available mixed IgG pool as an isotype control that stained nothing in our sections. Goblet cells were visualized using Alcian Blue Periodic acid schiff (AB/PAS) staining which was performed by the Augusta University histology core. Histological quantification and analysis was performed separately by two individuals blinded to treatments. At least 10 different sections containing approximately 8 crypts per section were counted for each mouse. The cGMP level in mouse colon mucosa was measured as previously described [23] using a cyclic GMP EIA kit (Cayman, Ann Arbor, MI).

### Intestinal transit assay

Intestinal transit assay was modified from previously reported methods [24,25]. Briefly, animals were fasted overnight before the experiments, with free access to water. Mice were gavaged with 100μl of charcoal meal (10% charcoal, 5% gum acacia in di-H_2_O) and placed individually into clean cages for monitoring. For total GI transit, mice were monitored every five minutes and the time from gavage of the charcoal meal to expulsion of a charcoal-marked fecal pellet was recorded [26]. For upper GI transit, mice were sacrificed ten minutes after administration of the charcoal meal and the small intestines were removed. The distance to the charcoal front and total length of small intestine from the stomach to the cecum were both measured to calculate upper transit. For acute drug studies, mice were gavaged with sildenafil or linaclotide one hour before the charcoal meal. Long term administration of the drugs started five days before the transit assay was to be performed. To determine the water content of the feces, individual fecal pellets were collected from each mouse and weighed. The pellets were dried in a 105°C oven for 24 hours then re-weighed. Water content was calculated using the following formula: ((Wet weight—dry weight)/wet weight)*100.

### DSS-induced inflammation recovery model

Six to eight week old C57/BL6 mice were treated with 3% DSS (m.w. 36000–50000; MP Biomedicals, Santa Ana, CA, USA) *ad libitum* in drinking water for five days. Mice were allowed to recover with normal drinking water for three weeks. Body weight was monitored daily throughout the study. After two weeks of recovery, mice were randomized into control and treatment groups. Long term sildenafil or linaclotide treatment was started during the last week of recovery. Acute sildenafil and linaclotide treatments were administered one hour prior to beginning the transit assay.

### Barrier permeability assay

Six to eight week old C57/BL6 mice were either untreated, or treated with 2% DSS for five days. The DSS-treated mice were randomized into two groups: control, and sildenafil. Mice were treated with sildenafil by oral gavage the night before and one hour prior to FITC administration. To measure barrier permeability, each group of mice, plus an untreated control group, were gavaged with 100μl of FITC-Dextran (100 mg/mL; 4kD; Sigma-Aldrich) after an overnight fast. 90 minutes after gavage, blood was collected from the submandibular vein from each mouse and serum was analyzed by fluorimetry to determine the concentration of FITC-dextran.

### Western blot analysis

Colon mucosa was scraped into ice-cold lysis buffer (50mM Tris-HCl, 1mM EDTA, 1% NP-40, 150mM NaCl) supplemented with protease and phosphatase inhibitor cocktail (CalBioChem). Tissue lysates were prepared and separated on PAGE gels. The lysates were subjected to immunoblot analysis using antibodies to the following antigens: Occludin (1:50000; AbCam), Cldn4 (1:1000; Santa Cruz), and β-actin (1:3000; Sigma).

### Statistical analyses

Results are presented as mean +/- SEM. Transit time and fecal water content were assessed with one-way analysis of variance (ANOVA) followed by Tukey’s multiple comparisons test. All other comparisons were performed with an unpaired student’s t-test. Differences in groups were considered significant if P < 0.05.

## Results

### PDE-5 inhibitors increase cGMP and regulate homeostasis in the mouse colon mucosa

**Fig 1:**
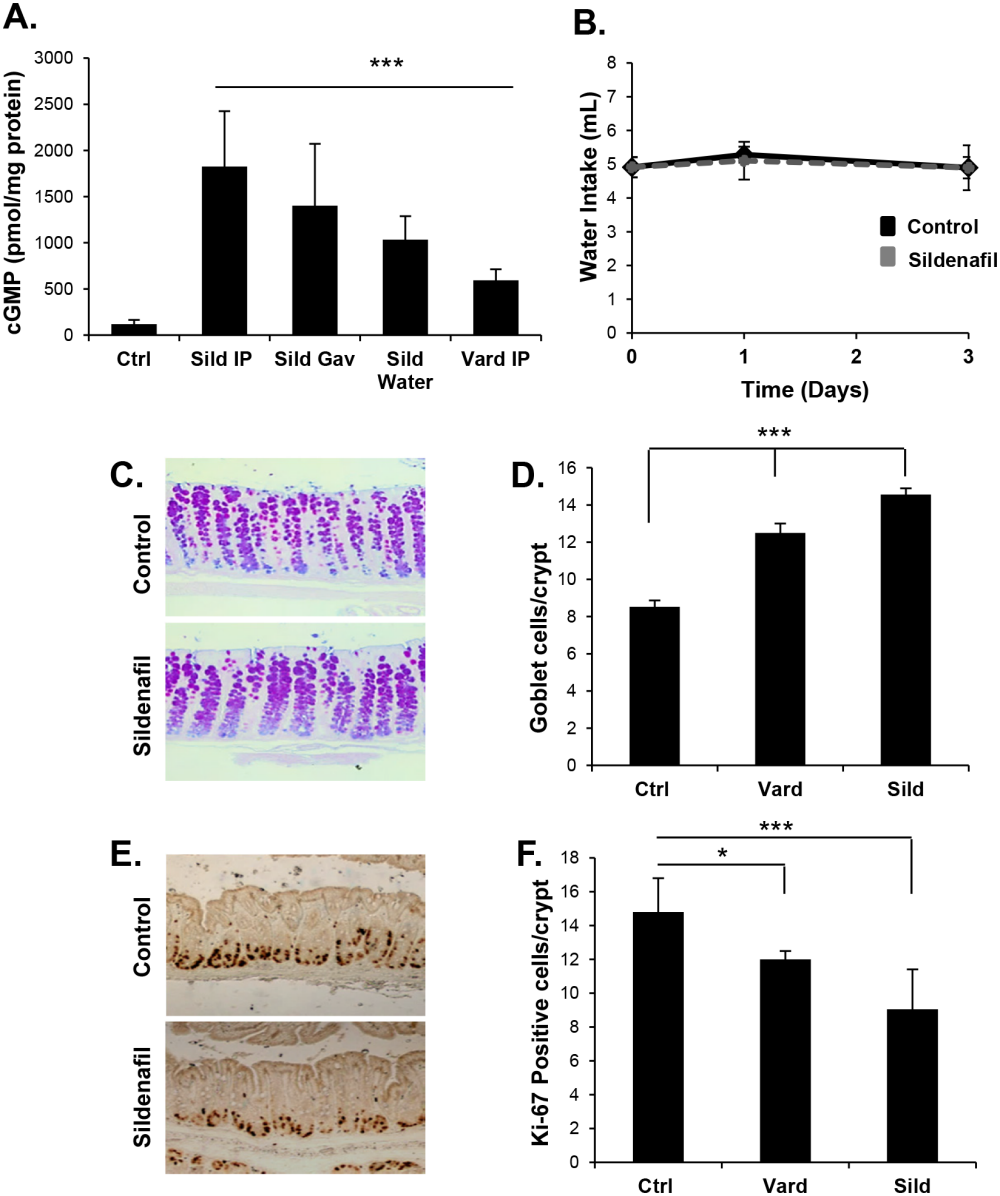
PDE-5 inhibitors increase cGMP and regulate homeostasis in the colon epithelium. (A) cGMP levels in the intestinal mucosa of untreated mice (Ctrl), or 5 h following gavage (Gav) or intraperitoneal injection (IP) of vardenafil or sildenafil, or mice provided sildenafil in water *ad libitum* (Sild Water) for 5 days. (B) Daily intake by mice provided water alone (Control) or water containing 35μg/ml sildenafil. (C, D) Quantification of goblet cells (ABPAS) and (E, F) Ki-67- positive cells in colon mucosa of CD-1 mice treated with vardenafil IP (vard) or sildenafil in drinking water (sild) for 5 days. Data are shown as means, error bars represent SEM, n = 6 (A, B), n = 3 (D, F). *P<0.05, ***P<0.001 by two-tailed Student’s t-test.

**Fig 2:**
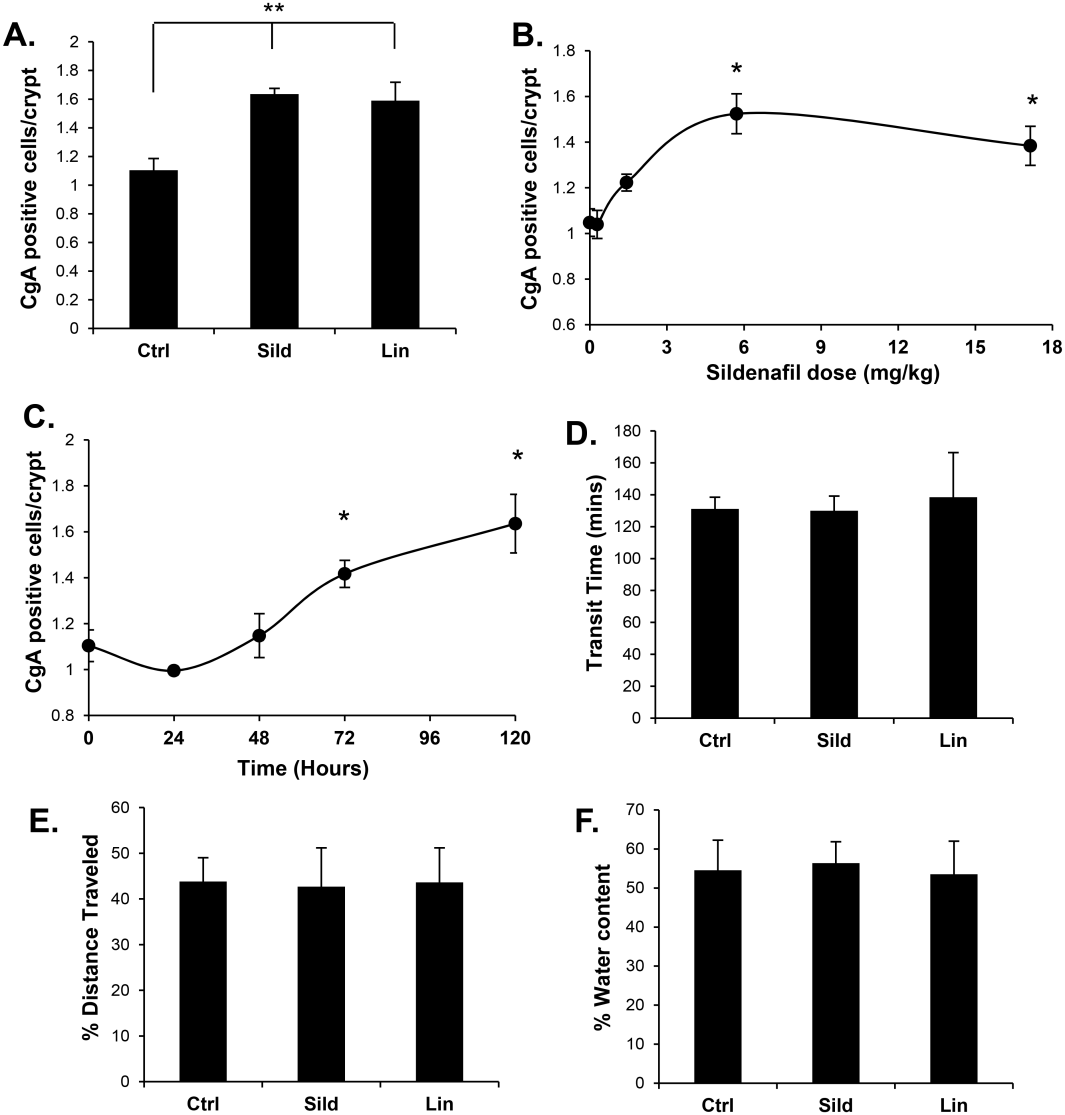
Sildenafil increases EE cell density in colon mucosa of healthy mice but has no effect on transit time or fecal water content. (A) Quantification of enteroendocrine cells (CgA) from stained tissue sections from colons of mice treated for five days with sildenafil, linaclotide, or untreated (Ctrl). (B) Quantification of enteroendocrine cells in colons from mice treated with various doses of sildenafil *ad libitum* in drinking water for five days. (C) CgA-positive cells over time following *ad libitum* sildenafil treatment. C57/BL6 mice were treated with sildenafil or linaclotide for five days prior to intestinal transit assay of (D) total transit or (E) upper intestinal transit. (F) Fecal pellets were collected from transit assays and measured for fecal water content. Data are shown as means, error bars represent SEM, n = 3 (A-C), n = 12 (D-E). *P<0.05, **P<0.005 by two-tailed Student’s t-test, each treatment group is compared to control (A, B) or time 0 (C).

Guanylin and uroguanylin are intestinal hormones that increase cGMP by binding and activating epithelial GC-C. With this endogenous cGMP-generating system, blocking cGMP degradation using PDE-5 inhibitors can increase cGMP levels. It was recently reported that increasing cGMP levels by intraperitoneal (IP) injection of the PDE-5 inhibitor vardenafil had profound effects on homeostasis in the colon epithelium [23]. In the present study, a single-dose of the PDE-5 inhibitor sildenafil administered by IP injection or oral gavage increased cGMP levels in the colon mucosa (15.2-fold and 11.7-fold respectively; p<0.001; Fig 1A). Furthermore, sildenafil administered to mice *ad libitum* in drinking water also increased cGMP levels in the colon (8.6-fold; p< 0.001; Fig 1A). At the doses used in our studies, inclusion of sildenafil in the water did not significantly affect daily water intake compared to controls (Fig 1B). In agreement with the previous study using vardenafil, sildenafil administration significantly increased the number of differentiated goblet cells in the colon epithelium and reduced the proliferating compartment (Fig 1C–1F). Sildenafil treatment also increased the CgA-positive enteroendocrine (EE) cell density, and this effect was saturable, with an EC_50_ of 1.5 mg/kg (Fig 2B).

Guanylin and GC-C knockout mice exhibit altered homeostasis in the colon, but the effects of exogenous GC-C ligand have not previously been examined. It was observed here that administration of linaclotide to mice had a similar effect on homeostasis as the PDE-5 inhibitors (Fig 2A). The biological effects of linaclotide occur luminally by activating GC-C expressed specifically in the intestinal epithelium. This contrasts with sildenafil, which is absorbed by the gut and has systemic effects on many tissues in addition to the colon epithelium. Because linaclotide and sildenafil have similar effects on gut homeostasis, it is likely that the effect of sildenafil in this tissue is due to its ability to increase cGMP in the colon epithelium by amplifying the effects of endogenous GC-C ligand, rather than indirectly by affecting other tissues. Since linaclotide is known to be beneficial in the treatment of IBS-C and CIC, it was hypothesized that sildenafil could also be used to increase bowel transit in constipation models. Since the therapeutic effects of linaclotide are thought to involve regulation of secretion and motility, it was of interest to determine whether sildenafil could affect bowel transit. However, it was found that neither sildenafil nor linaclotide treatment affected transit in healthy mice (Fig 2D and 2E). One of the most common side effects of linaclotide is diarrhea due to over-activation of GC-C and subsequent secretion. However, at the doses of the drugs used here, no change in fecal water content was observed (Fig 2F).

### Mice recovered from DSS-induced intestinal damage mimic post-infectious IBS

**Fig 3:**
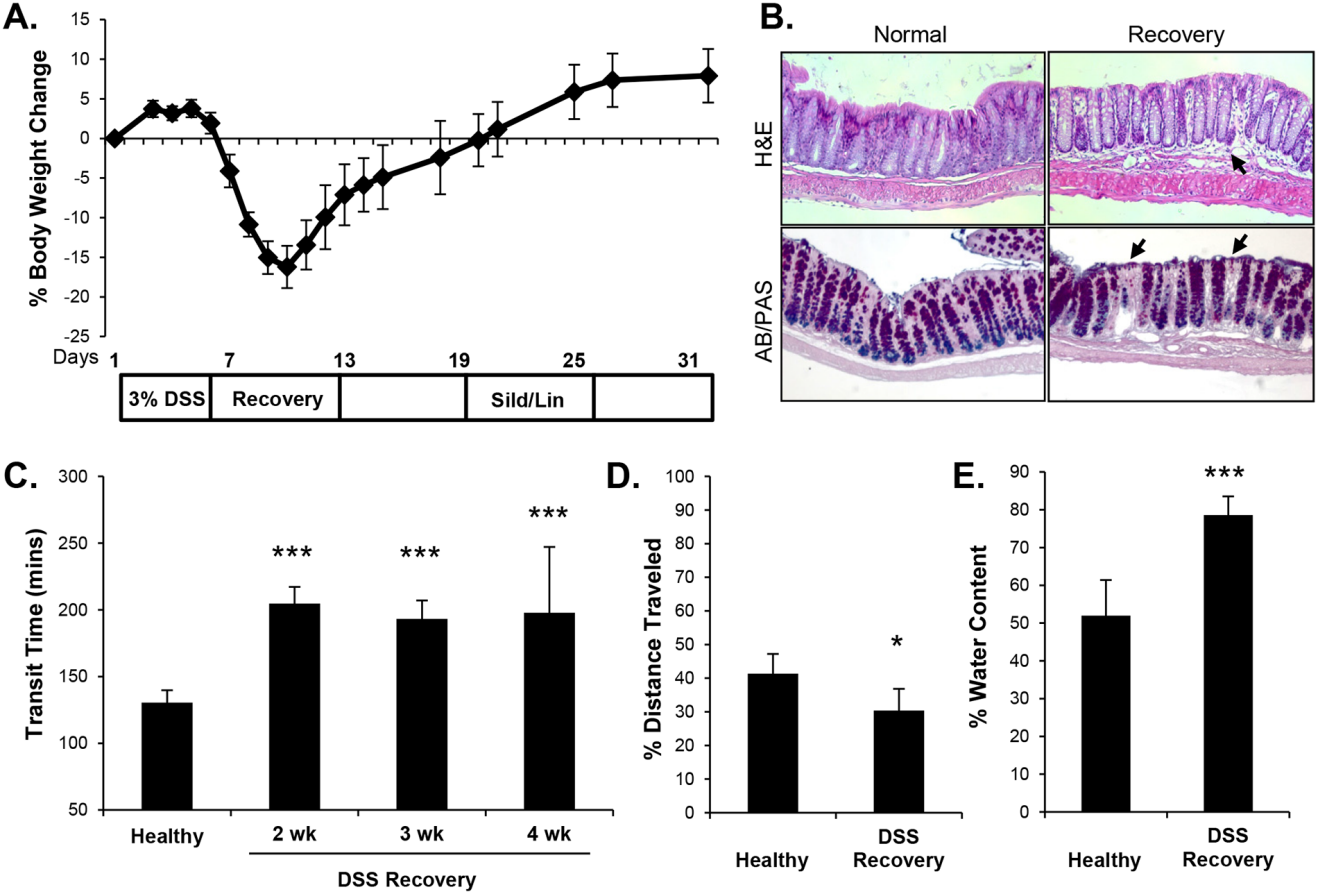
Mice recovering from DSS-induced inflammation have increased intestinal transit time and fecal water content. (A) 6–8 week old C57/BL6 mice were administered 3% DSS in drinking water for five days then allowed to recover for 3 weeks. (B) H&E and AB/PAS staining of a section of distal colon from a healthy mouse and a DSS-recovery mouse shows residual inflammation in recovery mice. Arrows show edema (upper panel) and crypt loss (lower panel) in the recovery animals. (C) Intestinal transit time for DSS-recovery mice for different recovery times. (D) Distance that a gavaged bolus of charcoal traveled in 10 minutes in mice recovered from DSS for three-weeks, compared to healthy mice. (E) Fecal water content of healthy and DSS-recovery mice. Data are shown as means, error bars represent SEM, n = 6 (C-E). *P<0.05, ***P<0.001 by two-tailed Student’s t-test.

As reported previously [27,28,29], administration of DSS (3%) to mice for five days in drinking water induced a well-characterized inflammatory response that was accompanied by loss of body weight (Fig 3A). Mice were allowed to recover for three weeks. At the time of the transit assay, body weight had normalized and disease symptoms were largely abrogated, suggesting that the mice had recovered from the DSS-induced intestinal inflammation. Histological analysis revealed that the recovered animals continued to exhibit evidence of a residual but stable disease marked by patches of edema and crypt loss (Fig 3B). Intestinal transit was significantly slower in mice recovered from DSS compared to healthy mice (Fig 3C). This change in transit was stable over several weeks (Fig 3C). Although damaging effects of DSS are more pronounced in the distal colon, the recovered mice exhibited slower upper intestinal transit as well as total intestinal transit (Fig 3D). Despite the reduced transit in the recovered mice, fecal water content was higher than matched control animals (Fig 3E).

### Sildenafil reduces IBS symptoms in a DSS-recovery mouse model

**Fig 4:**
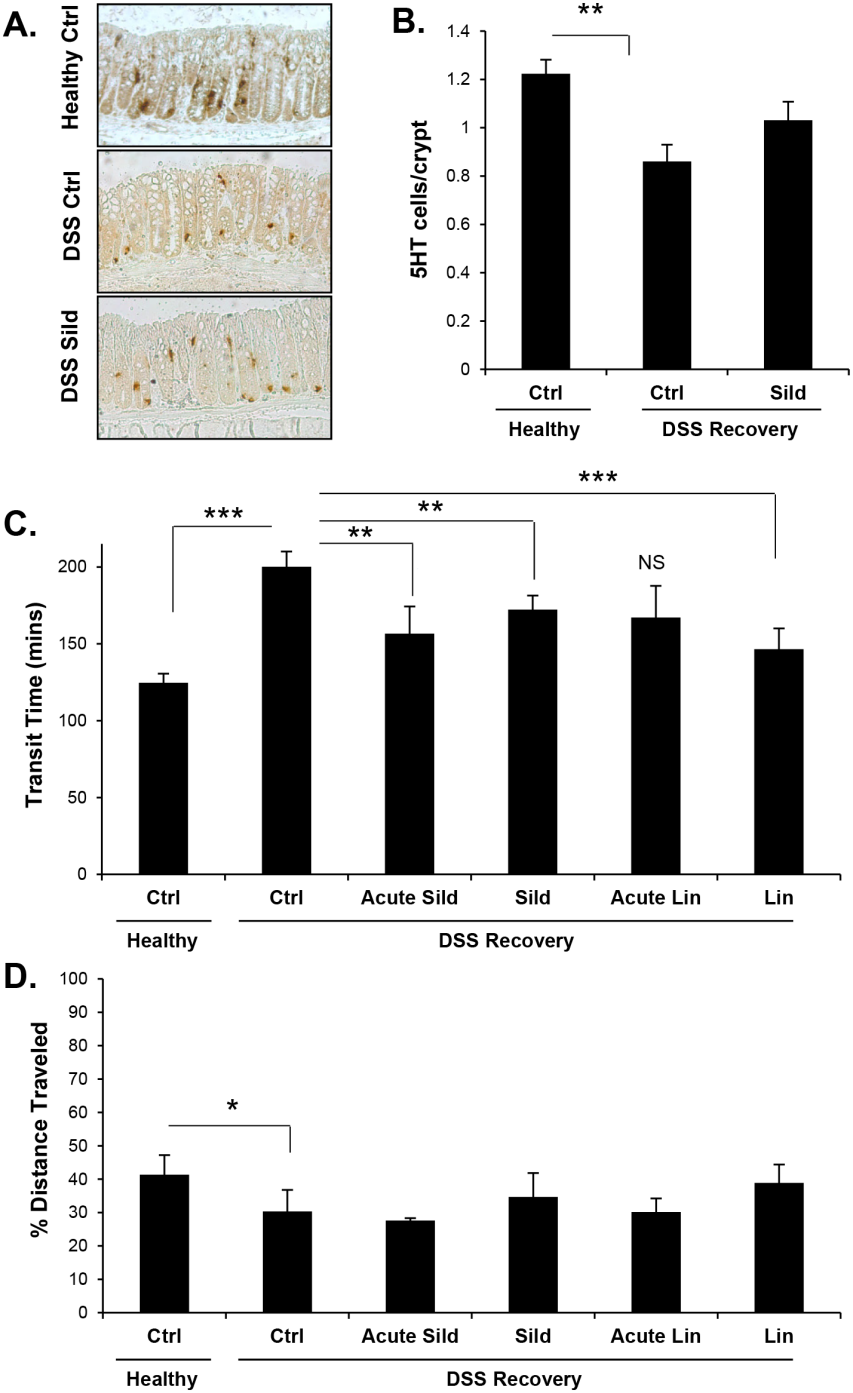
Sildenafil decreases intestinal transit time in a DSS-recovery model of IBS. (A,B) Quantification of 5-HT-positive cells in stained sections of colon from healthy mice, or those recovered from DSS treatment (DSS recovery). Mice were either untreated controls (Ctrl) or treated with sildenafil for five days (Sild). (C) Intestinal transit time and (D) distance traveled by a gavaged bolus of charcoal were measured in healthy and DSS-recovery mice. Mice were either untreated (Ctrl), or treated with sildenafil or linaclotide acutely (1 hr; Acute Sild, Acute Lin respectively) and 5-days or (Sild, Lin respectively). Data are shown as means, error bars represent SEM, n = 6 (B), n = 12 (C-D). *P<0.05, ** P<0.005, ***P<0.001 by one-way ANOVA and Tukey’s post hoc analysis.

The DSS-recovery mice exhibited reduced numbers of enterochromaffin (EC) cells in the colon epithelium compared to healthy mice (Fig 4A). Sildenafil treatment for five days reduced the deficit of these 5-HT-staining cells, and partially returned the density to that of healthy mice although it did not reach significance (Fig 4B). In addition, treatment of mice with either sildenafil or linaclotide reduced transit times in the DSS-recovery mice almost to the normal levels observed in healthy controls (Fig 4C). Notably, even an acute dose of either drug was effective at reducing intestinal transit time in this model. This result indicates that EC cell density is unlikely to contribute to the therapeutic effect of either drug in this model because the normalization of EC cell numbers required several days of sildenafil treatment. In addition, small intestinal transit time was unaffected by sildenafil treatment, suggesting that the regulation of transit in this model is largely due to increased cGMP in the colon (Fig 4D).

**Fig 5:**
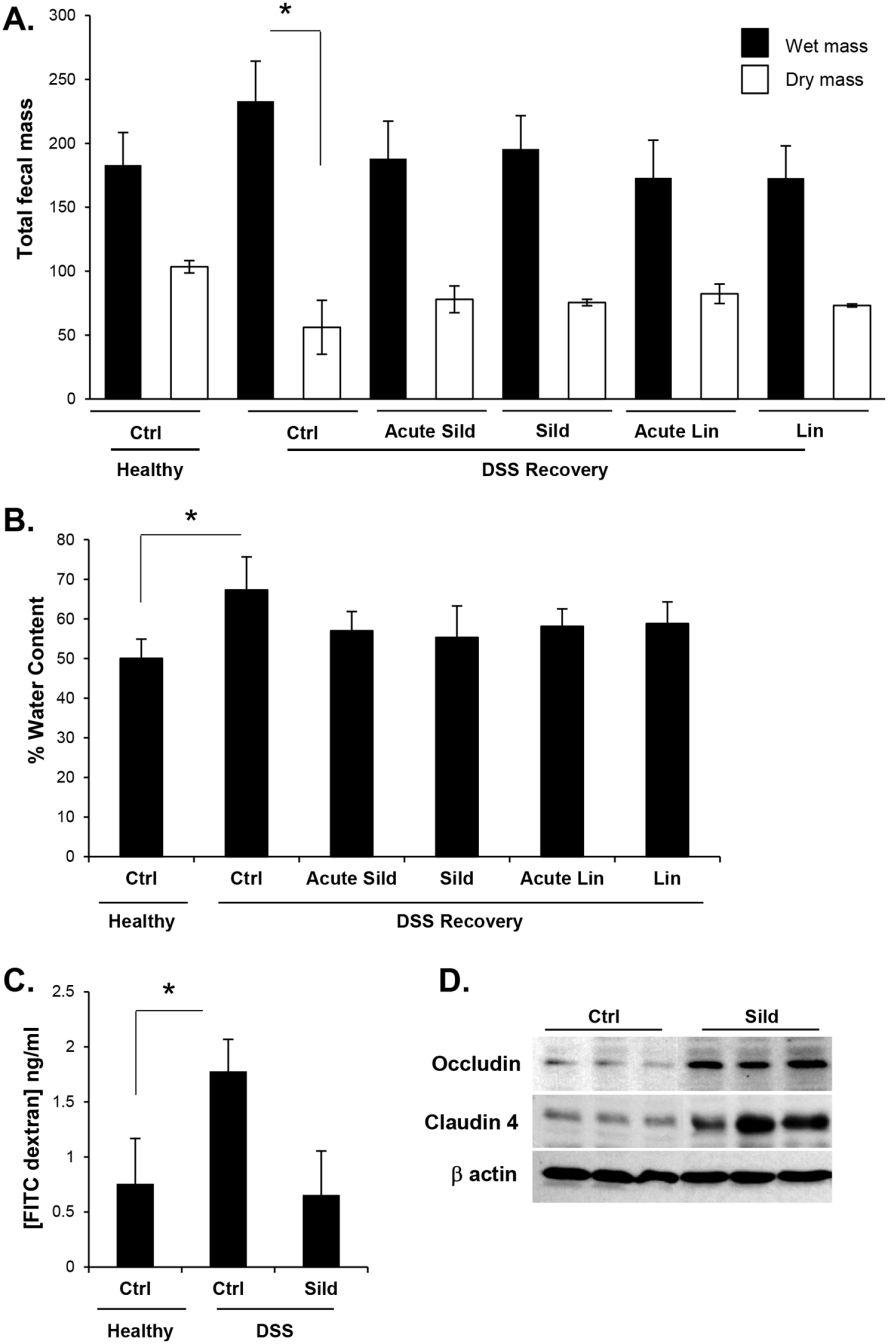
Sildenafil regulates fecal water content in DSS-recovery mice. Fecal pellets from healthy mice, or those recovered from DSS treatment (DSS recovery) were collected to measure (A) total wet and dry mass of pellets and (B) fecal water content. Mice were either untreated (Ctrl), or treated with sildenafil or linaclotide acutely (1 hr; Acute Sild, Acute Lin respectively) or 5-days (Sild, Lin respectively). (C) Mice were either provided water (healthy) or 3% DSS for 5 days (DSS). Barrier function was assessed using the FITC-dextran approach as described in the Materials and Methods. Mice exposed to DSS were either untreated (Ctrl) or treated with an acute dose of sildenafil (Sild). (D) Levels of tight junctional proteins claudin 4 and occludin were measured by western blot in mucosa collected from untreated mice (Ctrl) and mice treated sildenafil for 5 days (Sild). Data are shown as means, error bars represent SEM, n = 12 (A, B), n = 6 (C, D). *P<0.05, ***P<0.001 by one-way ANOVA and Tukey’s post hoc analysis.

Despite the slower transit of the DSS-recovered mice, the total fecal output was paradoxically slightly higher than untreated animals (Fig 5A). However, the total dry mass was significantly less in the diseased animals, indicating that the extra mass was attributed to water in the stool. Indeed, the average percentage of water per fecal pellet was higher in the diseased animals (Fig 5B). Administration of either sildenafil or linaclotide normalized the total fecal output and water content per fecal pellet. This effect was observed whether the drugs were given acutely (one hour prior to measurement), or longer term (5 days). Consistent with partial rescue of intestinal motility, sildenafil and linaclotide partially restored the total dry mass. These results suggest that residual inflammation in the colon of DSS-recovery mice more closely models PI-IBS, or low grade ulcerative colitis. Indeed, barrier-disruption by acute DSS treatment was restored by sildenafil treatment (Fig 5C). Knockout animals that are deficient in cGMP signaling components have a compromised intestinal barrier that has been suggested to be caused by reduced expression of junctional proteins [23,30,31]. Consistent with this idea, occludin and claudin 4 levels in the colon epithelium were increased by sildenafil treatment relative to untreated controls (Fig 5D).

### Sildenafil suppresses loperamide-induced constipation

**Fig 6:**
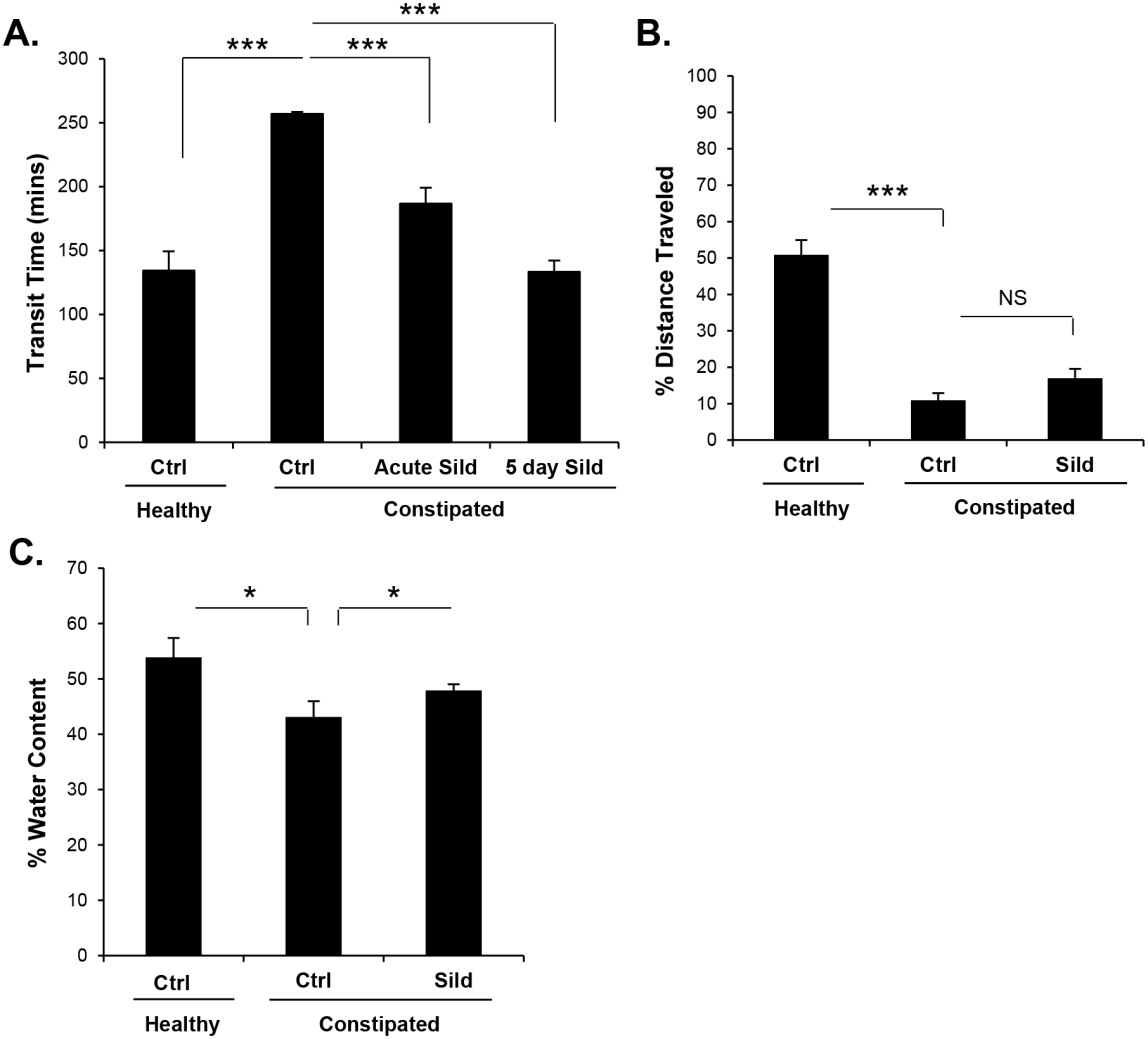
Sildenafil normalizes transit in loperamide-induced constipation model. Mice were either provided vehicle (healthy) or 10 mg/kg loperamide (constipated). (A) Total intestinal transit, (B) distance traveled by a gavaged bolus of charcoal, and (C) fecal water content were measured in untreated (Ctrl) or sildenafil treated (Sild) mice as indicated. Data are shown as means, error bars represent SEM, n = 12 (A-C). *P<0.05, ***P<0.001 by one-way ANOVA and Tukey’s post hoc analysis.

Linaclotide has been reported to relieve constipation by regulating both secretion and motility. It is unlikely that the normalization of transit conferred by sildenafil treatment of DSS-recovered mice was due to increased secretion, since the fecal wet weight was reduced in treated animals. This suggests that the regulation of intestinal transit by sildenafil treatment was more likely due to a direct effect on intestinal motility. To further explore this idea, the ability of sildenafil to normalize transit in loperamide treated animals was examined. Loperamide is a μ-opioid receptor agonist that acts on the myenteric plexus of the large intestine to decrease smooth muscle contractions and therefore models opioid-induced constipation [32]. In addition to regulating transit in the DSS-recovery model, sildenafil was also able to normalize transit in loperamide treated mice. Administration of loperamide to mice nearly doubled the intestinal transit time (Fig 6A). Both acute (one hour) and five day treatment of mice with sildenafil were able to block the loperamide-induced increase in intestinal transit time (Fig 6A). Loperamide also dramatically reduced transit in the small intestine, but this was not affected by sildenafil treatment (Fig 6B). As expected for mice administered loperamide, the reduced transit was accompanied by a decrease in fecal water content (Fig 6C). In contrast to the DSS-recovery model described above, but consistent with the increased transit, sildenafil administration increased fecal water content in the loperamide treated mice.

## Discussion

Irritable bowel syndrome is a significant health problem in the United States and there is a need for more effective treatments. The clinical success of the GC-C agonist linaclotide demonstrates the utility of cGMP elevation in the intestinal epithelium for the treatment of IBS-C and CIC patients. However, the mechanism of action is not fully understood and its contraindication in pediatric patients is an important limitation. An alternative approach to increase cGMP is by inhibition of cGMP degradation by blocking PDE-5. Indeed, administration of vardenafil to mice was recently shown to increase cGMP in the intestinal mucosa, and alter intestinal homeostasis [23]. Data shown here demonstrate that sildenafil, another PDE-5 inhibitor, can confer the same homeostatic changes when administered orally at doses used in humans to treat erectile dysfunction. In addition, for the first time we show that the GC-C agonist linaclotide can confer similar homeostatic changes in the colon. This supports the idea that the homeostatic effects of sildenafil in the colon result from augmentation of epithelial cGMP rather than from indirect effects resulting from systemic circulation of this drug. The mechanism underlying the effect of cGMP on intestinal homeostasis is poorly understood, but since both drugs required multiple days for maximal effect it is likely that gut turnover is important.

The similar effects of sildenafil and linaclotide on intestinal homeostasis prompted us to determine whether these drugs were also equally effective at treating constipation. There are no rodent models that fully mimic human disease, but a widely used approach involves allowing mice recover from bacterial infection, which mimics some aspects of post-infectious IBS (PI-IBS), [33,34]. Recovery from intestinal inflammation induced by chemical agents can also induce symptoms of IBS [35,36,37], and we demonstrate here that mice recovered from DSS-induced colitis exhibit slower intestinal transit, barrier dysfunction, and reduced EC cell density.

Treatment of DSS-recovered animals with either sildenafil or linaclotide increased intestinal transit in this model, but the mechanism of action downstream of cGMP is not clear. Sildenafil is absorbed systemically and has well-established relaxation effects on smooth muscle that might affect transit. Several studies have reported a reduction in esophageal contractile force by sildenafil treatment, particularly in patients with dysmotility disorders, but this effect is less pronounced in the normal esophagus and in the lower intestine [38,39,40,41,42]. Reduced muscular contractility cannot explain the results reported here because this would be expected to further increase transit time in our disease models, but as shown here, sildenafil reduced transit time. Moreover, sildenafil did not affect transit in healthy mice, nor did it affect transit in the upper gastrointestinal tract in the constipation models.

Altered serotonin function may play an important role in IBS; IBS-D patients often exhibit increased 5-HT signaling, and IBS-C patients typically have reductions in 5-HT signaling [4,43]. The DSS-recovered animals were shown here to be deficient in EC cells, and sildenafil treatment partially restored their number. Our initial hypothesis was that restoration of EC cell density contributed to the normalization of transit in these animals. However, this is unlikely because the effect of the cGMP-elevating drugs on transit was rapid, whereas the effect on EC cells required several days. Linaclotide was found to attenuate visceral pain in mice and in human IBS and CIC patients [19,44]. While the mechanism of analgesia remains unclear, it has been suggested that cGMP efflux by multi-drug resistance proteins (MRP4, 5) might directly suppress submucosal neuron excitability [44,45]. It is possible that this phenomenon might also mediate the effects of sildenafil on bowel transit in the work shown here. While linaclotide therapy has been reported to relieve constipation symptoms rapidly, improvement in visceral pain can take several days [18,46]. This suggests that cellular changes in the gut such as increased EC cell density might underlie the analgesic effects of linaclotide. This intriguing idea further suggests that sildenafil treatment might also confer analgesia in IBS patients, but whether the homeostatic changes observed in the mouse colon also occur in human patients treated with cGMP-elevating agents has not yet been examined.

Chronic, low-grade inflammation is common in IBS patients [47], and a defective intestinal barrier is likely to contribute to increased diarrhea and visceral hypersensitivity in IBS-D [43]. Several groups have shown that mice deficient in cGMP signaling components (GC-C and guanylin) have a dysfunctional intestinal barrier [30,31]. We demonstrate here for the first time that pharmacologically increasing cGMP in the colon epithelium can augment a dysfunctional epithelial barrier. Linaclotide is a secretagogue and has been reported to increase fecal water content in mice and humans [18,48,49]. This effect of cGMP in the gut has prevented GC-C agonist use in patients with IBS-D or IBD where diarrhea is a hallmark symptom. However, changes in fecal water content were not observed in healthy mice with the doses of sildenafil and linaclotide used here, indicating that low doses of either drug could affect homeostasis and barrier without over-activating secretion. This suggests that PDE-5 inhibitors and GC-C agonists might also be effective in treating PI-IBS or IBD patients, where the diarrhea is associated with a dysfunctional epithelial barrier rather than hypersecretion. However, the potential risks of systemic circulation of GC-C agonists in barrier-compromised patients will need further study.

In contrast to the damaging effects of DSS on the distal colon, loperamide does not affect intestinal barrier integrity, but works principally on the myenteric plexus to decrease tone of intestinal smooth muscles. We show here that loperamide increased transit time in mice, and as expected, this was associated with reduced fecal water content. Sildenafil was shown to normalize both transit and fecal water content in the loperamide model. While further study is necessary to identify the underlying mechanism, it is worthy to note that sildenafil did not affect small intestinal transit that was slowed by loperamide treatment. This suggests that the neuro-regulatory machinery that is responsive to cGMP regulation might be preferentially localized to the colon.

Taken together, the preclinical results shown here underscore the potential therapeutic value of PDE-5 inhibitors for the treatment of gastrointestinal disease. We demonstrated that sildenafil can normalize bowel transit in both a post-inflammatory and opioid-induced constipation models, suggesting possible benefit for human patients afflicted with IBS-C, CIC, and OIC. According to the prescribing information, preclinical studies revealed that linaclotide is lethal in juvenile mice due to increased fluid secretion leading to severe dehydration. The mechanism may be due to increased expression of GC-C in the intestinal epithelium, which would amplify the effect of exogenous ligand. It is likely that the increased GC-C expression is balanced by reduced endogenous ligand. This suggests that PDE-5 inhibitors are less likely to cause hypersecretion because they increase the basal cGMP level regardless of GC-C expression. Indeed, sildenafil is well-tolerated even long term in pediatric patients where it is used to treat pulmonary hypertension. Results described here suggest that sildenafil might also benefit pediatric patients suffering from CIC and IBS-C.

## Abbreviations

cGMP: 3’,5’cyclic guanosine monophosphate
CIC: chronic idiopathic constipation
DSS: dextran sulfate sodium
GC-C: guanylyl cyclase C
IBS: irritable bowel syndrome
IHC: immunohistochemistry
PDE-5: phosphodiesterase-5
Sild: Sildenafil
